# Supplementary material for: Metatranscriptome Profiling of a Specialized Microbial Consortium during the Degradation of Nixtamalized Maize Pericarp
Source: Microbiol Spectr. 2022 Jan 5;10(1):e02318-21. doi: 10.1128/spectrum.02318-21 (PMC8729791; doi:10.1128/spectrum.02318-21)

Supplemental file 1

Microbiology Spectrum

Metatranscriptome profiling of a specialized microbial consortium during the degradation of nixtamalized maize pericarp

José Germán Serrano-Gamboa [a], Carlos Abraham Fócil-Espinosa [a], Pedro J. Cabello-Yeves [b], Felipe H Coutinho [b], Rafael Antonio Rojas-Herrera [a] and Mónica Noel Sánchez-González [a*]

[a] Facultad de Ingeniería Química, Universidad Autónoma de Yucatán, Mérida, México.

[b] Evolutionary Genomics Group, Departamento de Producción Vegetal y Microbiología, Universidad Miguel Hernández, Alicante, Spain.

*Correspondence:
Name: Mónica Noel Sánchez-González
Email: monica.sanchez@correo.uady.mx

Supplemental material

Data Set S1. Functional annotation of predicted CAZymes encoded by PM-06 metatranscriptome. (S1A) General annotation of whole set of CDSs encoding putative CAZymes. (S1B) Classification of differentially expressed CAZymes by family number, bacterial genus, and target substrates.

Table S1. Predicted CAZymes and associated domains from PM-06 reference metatranscriptome targeting structural polysaccharides.

Figure S1. Recruitment plot of *Paenibacillus macerans* 8224 genome against 0 h metatranscriptome reads corresponding to 111.39 RPKG. Y-axis indicates percent of identity. X-axis indicates position in the genome (base pairs).

Figure S2. Heatmap showing the expression values log-CPM of all differentially expressed (21,000) transcripts of the PM-06 consortium across all samples (0, 4, 24 and 120 hours) along the degradation process of NMP.

Supplemental file 1

Table S1.

CAZy family and classification	Number of coding sequences
Hemicellulases	
GH8	1
GH10	5
GH11	1
GH26	5
GH28	3
GH31	7
GH39	1
GH42	6
GH43	22
Cellulases	
GH5	4
GH6	3
GH9	3
GH30	1
GH44	1
GH48	1
GH51	5
GH74	1
GH94	4
Deacetylases	
CE1	40
CE4	47
CE10	34
CE14	13
Oligosaccharide-degrading enzymes	
GH1	20
GH2	17
GH3	30

GH35	5
GH39	1
GH52	1
Oxidative enzymes	
AA3	7
AA10	3
Polysaccharide liases	
PL1	1
PL3	1
PL5	1
PL9	2
Carbohydrate-binding modules	
CBM3	11
CBM5	2
CBM6	4
CBM21	3
CBM32	8
CBM35	6
CBM41	2
CBM44	2
CBM48	7
CBM56	2
CBM59	1
CBM66	4
CBM67	4
Other domains	
S-layer homology (SLH)	12

Supplemental file 1

Figure S1.

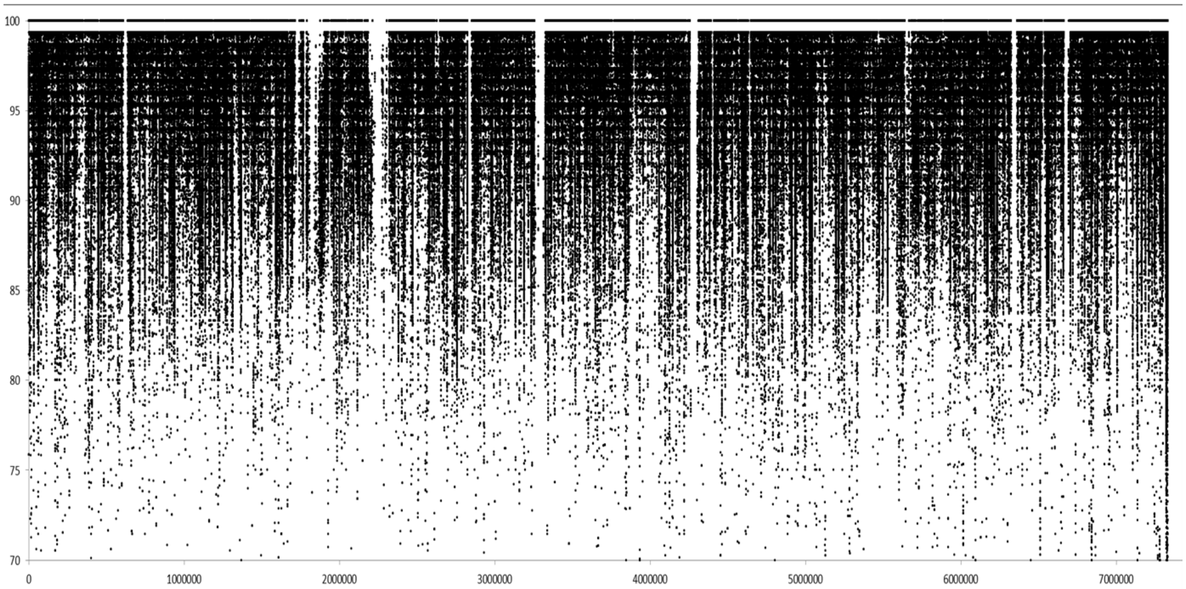

Supplemental file 1

Figure S2.

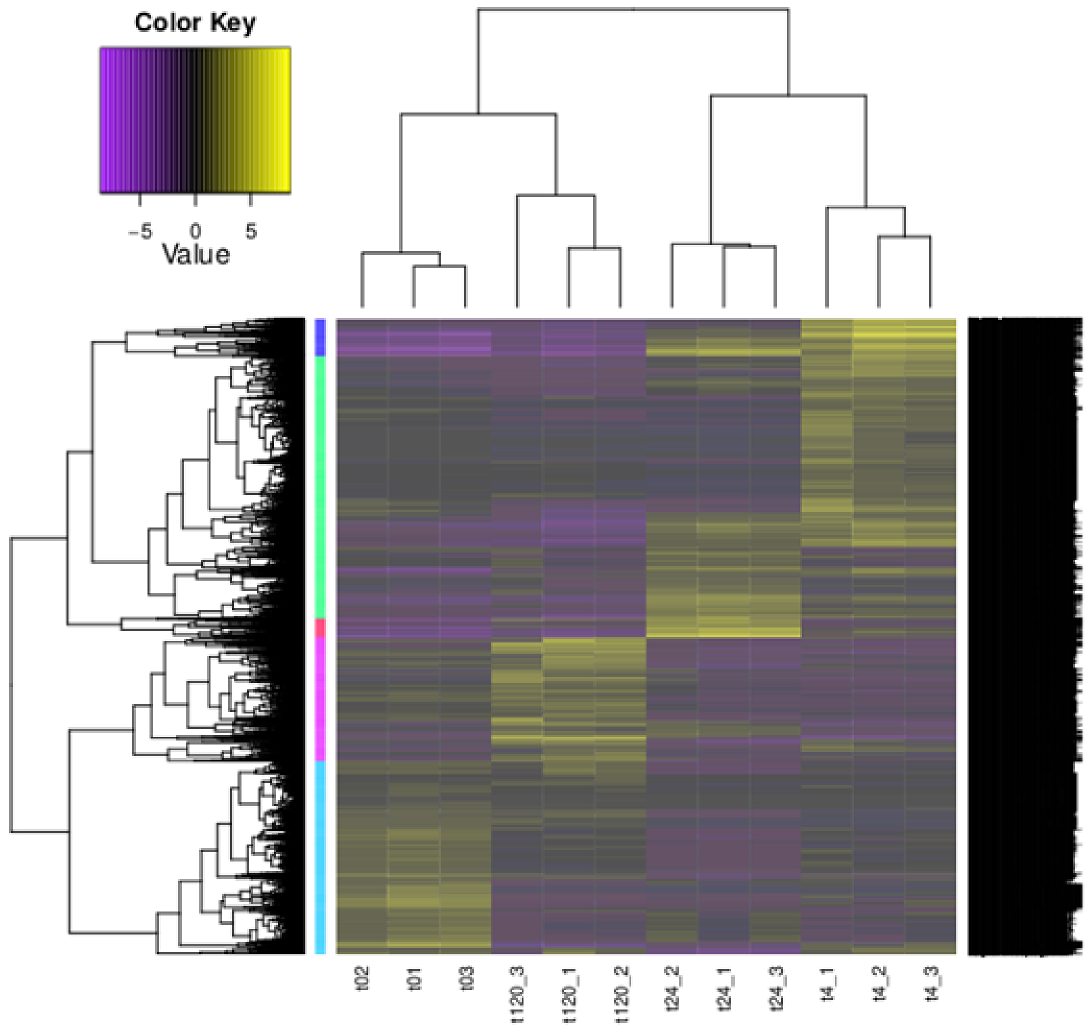

Supplement: SUPPLEMENTAL FILE 2 — Supplemental material. Download SPECTRUM02318-21_Supp_1_seq12.pdf, PDF file, 0.7 MB [file spectrum02318-21_supp_1_seq12.pdf]
